# Beneficial properties of lactic acid bacteria naturally present in dairy production

**DOI:** 10.1186/s12866-018-1356-8

**Published:** 2018-12-19

**Authors:** Monique Colombo, Nathália P. A. Castilho, Svetoslav D. Todorov, Luís Augusto Nero

**Affiliations:** 1 Departamento de Veterinária, Universidade Federal de Viçosa. Campus UFV, Viçosa, MG 36570-900 Brazil; 2 Departamento de Veterinária, Campus UFV Universidade de São Paulo, Faculdade de Ciências Farmacêuticas, 36570-900, Viçosa, MG Brazil; 3 Departamento de Alimentos e Nutrição Experimental, Av. Prof. Lineu Prestes, 580, Bloco 1, Cidade Universitária, São Paulo, SP 05508-000 Brazil

**Keywords:** Beneficial potential - lactic acid bacteria, Dairy

## Abstract

**Background:**

Consumers are increasingly demanding for natural and beneficial foods, in order to improve their health and well-being. Probiotics play an important role in such demand, and dairy foods are commonly used as vehicles for such bacteria, represented predominantly by lactic acid bacteria. Due to consumers demand, food industry is constantly looking for novel bacterial strains, leading to studies that aims the isolation and characterization of their beneficial features. This study aimed to characterize the naturally occurring lactic acid bacteria obtained from a dairy environment, in order to assess their potential use as probiotics.

**Results:**

Preliminary screening and PCR analysis, based on 16S rRNA sequencing, were applied to select and identify 15 LAB strains from the genera *Lactobacillus* (*n* = 11), *Pediococcus* (*n* = 2) and *Weissella* (n = 2). All strains showed resistance to low pH and the evaluated bile salt concentrations in vitro. The API ZYM test characterized the enzymatic activity of the strains, and a high β-galactosidase activity was observed in 13 strains. All strains presented resistance to simulated gastric (3 h) and intestinal (4 h) conditions in vitro, the ability to auto- and co-aggregate with indicator microorganisms and a high cell surface hydrophobicity. Most of the strains were positive for *map* and *EFTu* beneficial genes. All strains exhibited strong deconjugation of bile salts in vitro and all assimilated lactose.

**Conclusions:**

The phenotypes exhibited in vitro and the presence of beneficial genes revealed the beneficial potential of the studied strains, demanding further analyses in a food matrix and in vivo to allow the development of a functional product, with health-related properties.

**Electronic supplementary material:**

The online version of this article (10.1186/s12866-018-1356-8) contains supplementary material, which is available to authorized users.

## Background

Probiotics are defined as living organisms that benefit consumer health when ingested in adequate concentration by the World Gastroenterology Organization [1]. The rise in probiotic product consumption is fueled by the increasing trend in consumers seeking products that improve life quality. Health and well-being are directly linked to good nutrition, physical activity and lifestyle [2]. In this context, probiotic strains embrace the concept of good nutrition by assisting with health maintenance, through the prevention, control and treatment of diseases [3].

More research is needed to isolate and characterize beneficial bacteria with probiotic potential, to meet the consumer demand. Dairy production systems are important sources of beneficial strains, and fermented products are still the main sources of probiotic bacteria [4]. Lactic acid bacteria (LAB) are one of the most significant groups of probiotic organisms, commonly used in fermented dairy products. Among other benefits, these microorganisms can enhance lactose digestion, stimulate the immune system, and prevent and treat diarrhea [5].

Thus, the current study aimed to explore the dairy production environment as a source of LAB strains with probiotic potential.

## Methods

### Samples

Raw milk, swabs from cow and goat saliva and vaginal mucosa, ruminal boluses, consumption water and silage were collected from dairy farms (goat and cattle) located in the Universidade Federal de Viçosa, Viçosa, Minas Gerais state, Brazil, with conventional management and production destined to dairy processing. The samples were obtained after agreement of the responsible sector for managing these farms (Animal Science Department, Universidade Federal de Viçosa) and kept refrigerated before the following analyses.

### LAB isolation and characterization

All samples were ten-fold diluted with 0.85% NaCl (*w*/*v*). Selected dilutions were pour-plated in Man, Rogosa and Sharpe agar (MRS, Oxoid Ltd., Basingstoke, England) and MRS supplemented with 10 mg/L vancomycin (Sigma–Aldrich, St. Louis, MO, USA) for LAB enumeration, according to M Colombo, AEZ Oliveira, AF Carvalho and LA Nero [6]. Representative colonies were selected (10% of the observed count) and tested for Gram stain and catalase reaction. The preliminary LAB characterized isolates (Gram-positive and catalase-negative) were freeze-dried and stored at − 20 °C. Further microbiological analyses were conducted, as described in the sections below.

### Gastric pH resistance

Bacterial cells were grown overnight and prepared for the gastric pH resistance test, according to AA Argyri, G Zoumpopoulou, KG Karatzas, E Tsakalidou, GE Nychas, EZ Panagou and CC Tassou [7]. Resistance, assessed in triplicate, was evaluated by viable colony counts on MRS agar after incubation at 37 °C for 0 and 3 h, reflecting the time spent by food in the stomach. The resistance to low pH was performed as described by SD Todorov, DN Furtado, SMI Saad, E Tome and BDGM Franco [8], with some modifications. The isolates were grown at 37 °C in MRS broth adjusted to pH 2.0, 2.5 and 3.0 with HCl until the cell density reached 3 × 10^7^ CFU/mL. All tests were conducted in sterile flat-bottom 96-well microtiter plates (Thermo Scientific, Waltham, MA, USA). In order to compare the count with the absorbance reading, optical density (OD) measurements were recorded at 650 nm at zero time and after incubation at 37 °C for 3 h (aerobic condition), using a microtiter plate reader (BioTek Instruments, Inc., Winooski, VT, USA). Cultures grown in MRS broth corrected to pH 7.2, served as the control.

### Bile resistance

After preparing the bacterial inoculum [7], the resistance to bile salts was assessed, based on SD Todorov, DN Furtado, SMI Saad, E Tome and BDGM Franco [8], with some modifications. The isolates were grown at 37 °C in MRS broth containing 0.5 and 3% (*w*/*v*) bile salts (Sigma), using 96-well microtiter plates, as described above. The OD readings were recorded at zero time and after incubation at 37 °C for 4 h. Cultures grown in MRS broth without bile served as the control.

### Molecular identification

DNA of 82 selected isolates was extracted using a ZR Fungal/Bacterial DNA kit (Zymo Research, Irvine, CA, USA), and the DNA concentrations determined using NanoDrop (Thermo Scientific). Repetitive-element PCR and gel electrophoresis were performed according to the protocol described by B Dal Bello, K Rantsiou, A Bellio, G Zeppa, R Ambrosoli, T Civera and L Cocolin [9], using the single primer GTG_5_ (Additional file 1: Table S1). The electrophorezed gels were stained with Gel Red (Biotium, Inc., Hayward, CA, USA) and the bands were visualized and documented using an ultraviolet transilluminator (LPIX, Loccus Biotecnologia, São Paulo, SP, Brazil). Further differentiation of the LAB strains was achieved by random amplification of polymorphic DNA, as detailed by SD Todorov, M Wachsman, E Tomé, X Dousset, MT Destro, LMT Dicks, BDG de Melo Franco, M Vaz-Velho and D Drider [10]. Taxonomic identification was confirmed by sequencing of PCR-amplified 16S rRNA using the universal pair of primers 8F and 1512R [11]. Sequencing of the amplicons was done at the Center for Human Genome Studies, Institute of Biomedical Sciences, University of São Paulo (São Paulo, SP, Brazil). Obtained sequences were compared to reference sequences in GenBank, using the basic local alignment search tool (BLAST).

### Detection of enzymatic activity

The enzymatic activity of each of the selected isolates was established, according to the API ZYM Kit (bioMérieux, Marcy-l’Étoile, France) manufacturer’s manual. The following enzymes were tested: alkaline phosphatase, esterase, esterase/lipase, lipase, leucine arylamidase, valine arylamidase, cysteine arylamidase, trypsin, α-chymotrypsin, acid phosphatase, naphthol-AS-BI-phosphohydrolase, α-galactosidase, β-galactosidase, β-glucuronidase, α-glucosidase, β-glucosidase, *N*-acetyl-β-glucosaminidase, α-mannosidase and α-fucosidase.

### Resistance to simulated gastric and intestinal conditions

The tolerance of the selected strains to gastric and intestinal conditions was evaluated through an in vitro model described by KMO Santos, ADS Vieira, FCA Buriti, JCF Nascimento, MES Melo, LM Bruno, MF Borges, CRC Rocha, ACS Lopes and BDGM Franco [12]. The assay was performed three times for each strain, and the enumeration was done in duplicate. The survival rate (SR) of strains after gastric and enteric simulation were calculated using the equation: SR (%) = [log CFU *N*/log CFU *N*_*0*_] ×  100 [13], where *N*_*0*_ and *N* are the populations before and after the assay, respectively. The mean counts of log populations were compared by analysis of variance (ANOVA) and Tukey’s test (*p* < 0.05) using XLSTAT 2016.01.26192 (Addinsoft, New York, NY, USA).

### Aggregation and co-aggregation properties

Aggregation abilities of the 15 selected LAB were tested using the method proposed by SD Todorov, DN Furtado, SMI Saad, E Tome and BDGM Franco [8] and Y Zhang, L Zhang, M Du, H Yi, C Guo, Y Tuo, X Han, J Li, L Zhang and L Yang [14]. Auto-aggregation was determined using the following equation: % auto-aggregation = [(OD_0_ – OD_60_) / OD_0_] × 100. OD_0_ and OD_60_ refer to the initial OD and the OD determined at 60 min, respectively.

For evaluation of co-aggregation, the 15 selected strains were grown in 10 mL of MRS and *Listeria monocytogenes* Scott A, *Enterococcus faecalis* ATCC 19443 and *Lactobacillus sakei* ATCC 15521 in brain heart infusion (Oxoid) and MRS (Oxoid), respectively, at 37 °C [8]. Co-aggregation was calculated using the following equation: % co-aggregation = [(OD_0−_ OD_60_) / OD_60_] × 100. OD_0_ refers to the initial OD, taken immediately after the relevant strains were paired. OD_60_ refers to the OD of the supernatant at 60 min. Experiments were conducted in triplicate on two separate occasions.

The bacterial adhesion to hydrocarbons was tested as described by RJ Doyle and M Rosenberg [15], using 15 selected LAB strains. The percentage hydrophobicity was calculated as follows: % hydrophobicity = [(OD_580_ reading 1 – OD_580_ reading 2) / OD_580_ reading 1] × 100. Experiments were conducted in triplicate.

Finally, DNA obtained from the selected strains was analyzed by PCR for the presence of genes (Additional file 1: Table S1) related to the adhesion characteristics. The target genes included *EF2380, EF2662, prgB, EF1249* [16], *map, mub* and *EFTu* [17].

### Bile salt deconjugation

The selected strains were evaluated by their ability in deconjugate bile salts, as described by KMO Santos, ADS Vieira, FCA Buriti, JCF Nascimento, MES Melo, LM Bruno, MF Borges, CRC Rocha, ACS Lopes and BDGM Franco [12], using sodium salts of taurocholic acid (TC), taurodeoxycholic acid (TDC), glycocholic acid (GC) and glycodeoxycholic acid (GDC) (all from Sigma–Aldrich), in two repetitions and in duplicate.

### β-Galactosidase activity

The assay described by KMO Santos, ADS Vieira, FCA Buriti, JCF Nascimento, MES Melo, LM Bruno, MF Borges, CRC Rocha, ACS Lopes and BDGM Franco [12] was considered to assess the β-galactosidase activity of the selected strains, using sterile filter paper discs impregnated with *o*-nitrophenyl-β-D-galactopyranose (ONPG discs, Fluka, Buchs, Switzerland), in two repetitions and in duplicate.

### Lactose assimilation

The ability of LAB strains to metabolize lactose was tested by the strains cultivation in modified MRS, with 2% lactose as the single carbon source, at 37 °C for 24 h. Cultures obtained under the same conditions but on MRS with 2% glucose as the carbon source were used as the controls. The growth of the strains was estimated by viable cell counts, after plating 10-fold serial dilutions on MRS agar medium [18]. The mean counts of log populations were compared by ANOVA (*p* < 0.05) using XLSTAT 2016.01.26192 (Addinsoft).

## Results

### Screening

A panel of 500 isolates was obtained from dairy environment samples, selected due to the results from the initial survival tests on pH and bile, being 394 both Gram-positive and catalase-negative. The final stage before conducting the proper assays for beneficial activity was survival in extreme conditions within the gastrointestinal tract (GIT); results were considered positive for growth in MRS broth at low pH and a high concentration of bile salts. After these screening tests, from 394 isolates, 82 were able to resist pH 2.0 and 3% bile (in MRS broth) and were selected and molecularly fingerprinted. Results showed that from the 82 tested strains, 15 could be considered unique, so were chosen for taxonomical identification by sequencing of the PCR-amplified 16S rRNA. *Lactobacillus casei* MSI1, *L. casei* MSI5, *L. acidophilus* MVA3, *L. harbinensis* MSI3, *L. plantarum* MLE5, *L. plantarum* MSI2 and *Pediococcus acidilactici* MSI7 were isolated using MRS, and *L. casei* MRUV1, *L. casei* MRUV6, *L. nagelli* MSIV4, *L. harbinensis* MSIV2, *L. fermentum* SIVGL1, *P. pentosaceus* MLEV8, *Weissella paramesenteroides* MRUV3 and *W. paramesenteroides *MSAV5 were isolated using MRS-V.

**Fig. 1:**
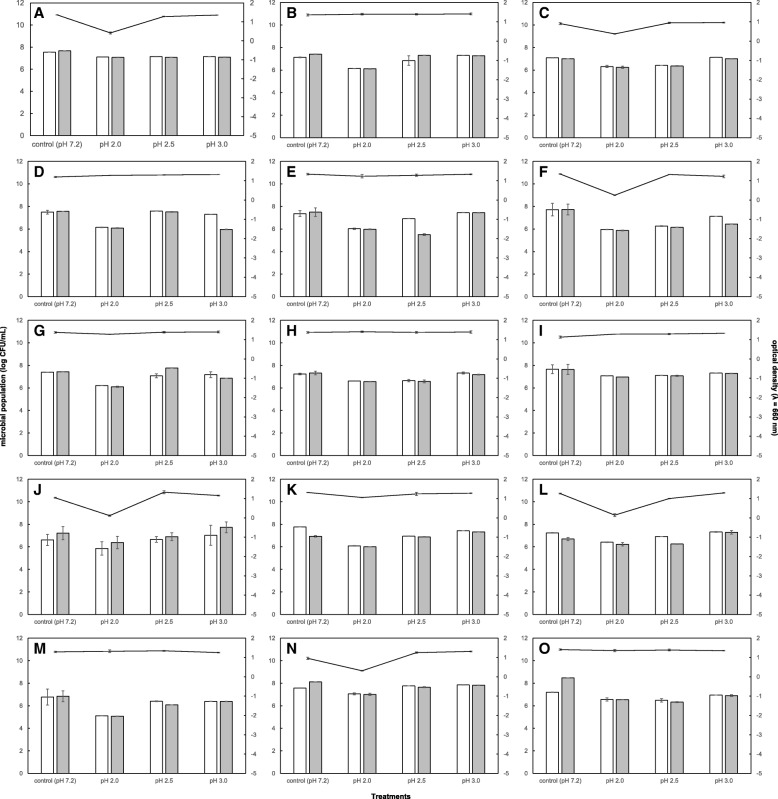
Resistance of lactic acid bacteria isolated from dairy production with beneficial potential to effect of low pH as determined at 0 h and 3 h in non-growing conditions (results are expressed as log_10_ CFU/mL) and growth of LAB for 18 h after been exposed to the effect of low pH for 3 h (results are expressed as OD 650 nm determined on microplate reader). **a** *Lactobacillus casei* MSI1; **b** *L. casei* MSI5; c *L. casei* MRUV1; **d** *L. casei* MRUV6; **e** *L. acidophilus* MVA3; **f** *L. nagelli* MSIV4; **g** *L. harbinensis* MSI3; **h** *L. harbinensis* MSIV2; **i** *L. fermentum* SIVGL1; **j** *L. plantarum* MLE5; **k** *L. plantarum* MSI2; **l** *Pediococcus pentosaceus* MLEV8; **m** *P. acidilactici* MSI7; **n** *Weissella paramesenteroides* MRUV3; **o** *W. paramesenteroides* MSAV5. The white bars represent the counts of the LAB strains at the initial time (zero) and the grey bars represent the counts after 3 h incubated in the different pH treatments. The solid line represents the values of optical density in the different pH treatments

**Fig. 2:**
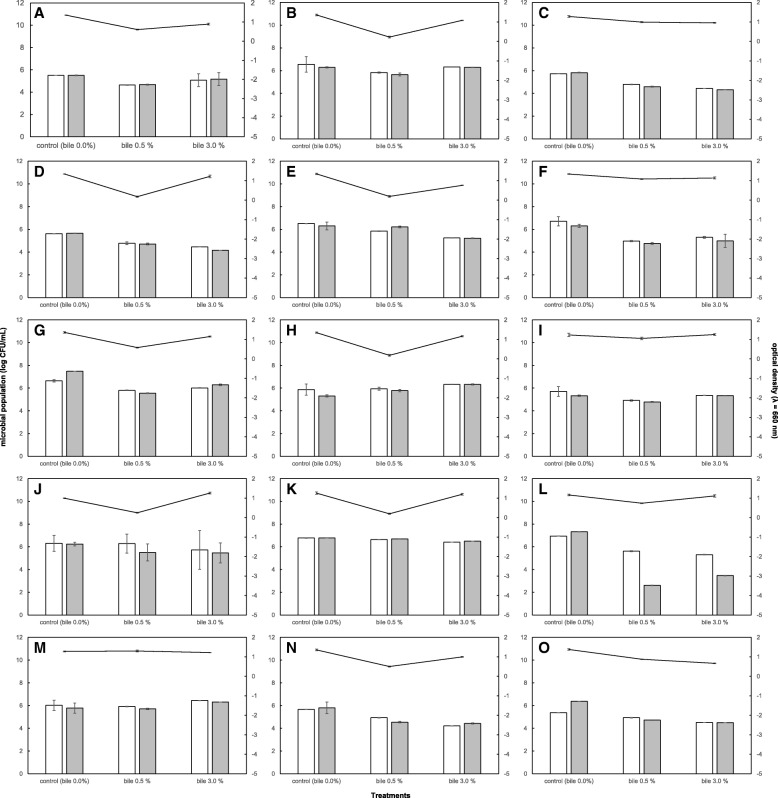
Resistance of lactic acid bacteria isolated from dairy production with beneficial potential to effect of bile salts as determined at 0 h and 4 h in non-growing conditions (results are expressed as log_10_ CFU/mL) and growth of LAB for 18 h after been exposed to the effect of bile salts for 4 h (results are expressed as OD 650 nm determined on microplate reader). **a**: *Lactobacillus casei* MSI1; **b**: *L. casei* MSI5; **c**: *L. casei* MRUV1; **d**: *L. casei* MRUV6; **e**: *L. acidophilus* MVA3; **f**: *L. nagelli* MSIV4; **g**: *L. harbinensis* MSI3; **h**: *L. harbinensis* MSIV2; **i**: *L. fermentum* SIVGL1; **j**: *L. plantarum* MLE5; **k**: *L. plantarum* MSI2; **l**: *Pediococcus pentosaceus* MLEV8; **m**: *P. acidilactici* MSI7; **n**: *Weissella paramesenteroides* MRUV3; **o**: *W. paramesenteroides* MSAV5. The white bars represent the counts of the LAB strains at the initial time (zero) and the grey bars represent the counts after 4 h incubated in the different bile treatments. The solid line represents the values of optical density in the different bile treatments

Resistance to gastric pH and high bile concentrations are key features for cultures to be able to resist the unfavorable conditions of the GIT. As shown in Figs. 1 and 2, the 15 selected LAB strains had a high SR under the treatment conditions. Figure 1 illustrates that the tested strains were able to survive the gastric pH. None of the studied cultures presented a population decrease higher than 1 log. This behavior was also reflected in the OD changes (Fig. 1). *L. casei* MSI5, *L. casei* MRUV6, *L. acidophilus* MVA3, *L. harbinensis* MSI3, *L. harbinensis *MSIV2, *L. fermentum* SIVGL1, *L. plantarum* MSI2, *P. acidilactici* MSI7 and *W. paramesenteroides* MSAV5 cultures displayed higher SRs compared to the other strains. Bile salts, at various concentrations, affected the survival of the tested strains. Among the 15 LAB strains selected for their good resistance to low pH, all strains exhibited reasonably good bile tolerance after incubation in the presence of bile salts for 4 h (Fig. 2). The changes in OD supported the findings (Fig. 2). The strains that exhibited a higher sensitivity to treatment with bile salts were *L. casei* MSI1, *L. casei* MRUV1, *L. acidophilus* MVA3 and *W. paramesenteroides* MSAV5.

The API ZYM kit test results for the enzymatic activity patterns of the assessed strains are presented in Table 1. All tested strains were positive for leucine arylamidase, acid phosphatase and naphthol-AS-BI-phosphohydrolase. *L. harbinensis* MSIV2 were positive for production of 17 enzymes, as part of the API ZYM kit, and negative for α-mannosidase and α-fucosidase. Lipase, trypsin and β-glucuronidase activities were absent in most of the strains, and α-mannosidase and α-fucosidase activities were missing in all 15 tested strains.

**Table 1:** Enzymatic profile of the studied 15 lactic acid bacteria (LAB) strains determined by APIZYM test

LAB		Alkaline phosphatase	Esterase	Esterase lipase	Lipase	Leucine arilamidase	Valine arilamidase	Cistine arilamidase	Trypsin	α- chymotrypsin	Acid phosphatase	Naphthol phosfohydrolase	α- galactosidase	β- galactosidase	β- glucuronidase	α- glucosidase	β- glucosidase	N-acetyl-β-glucosaminidase	α- manosidase	α- fucosidase
*L. casei*	MSI1	–	+	+	–	+	+	–	–	–	+	+	+	+	–	+	+	–	–	–
	MSI5	+	+	+	–	+	+	+	–	+	+	+	–	+	–	+	+	+	–	–
	MRUV1	–	–	–	+	+	+	+	–	–	+	+	–	+	–	–	+	+	–	–
	MRUV6	–	–	–	–	+	+	+	–	–	+	+	–	+	–	–	+	–	–	–
*L. acidophilus*	MVA3	–	+	+	–	+	–	+	–	+	+	+	+	–	–	–	–	–	–	–
*L. nagelli*	MSIV4	–	+	+	–	+	–	–	–	–	+	+	+	–	–	–	–	–	–	–
*L. harbinensis*	MSI3	+	+	+	+	+	+	+	+	–	+	+	–	+	–	+	+	+	–	–
	MSIV2	+	+	+	+	+	+	+	+	+	+	+	+	+	+	+	+	+	–	–
*L. fermentum*	SIVGL1	–	–	–	–	+	+	+	–	–	+	+	–	+	–	–	+	+	–	–
*L. plantarum*	MLE5	+	+	+	–	+	+	+	–	+	+	+	–	+	–	+	–	–	–	–
	MSI2	–	+	+	–	+	+	+	–	–	+	+	–	+	–	+	+	+	–	–
*P. pentosaceus*	MLEV8	–	+	+	–	+	+	–	–	+	+	+	+	+	–	–	–	–	–	–
*P. acidilactici*	MSI7	–	–	–	–	+	+	+	+	–	+	+	–	+	–	–	+	+	–	–
*W. paramesenteroides*	MRUV3	–	–	–	–	+	+	+	–	–	+	+	–	+	–	–	+	+	–	–
	MSAV5	–	+	+	–	+	+	+	–	+	+	+	+	+	–	+	+	–	–	–

### Beneficial properties in vitro

Examination of the survival during in vitro simulation of the gastric and intestinal phases revealed the tested strains were able to survive and even multiply under the gastric phase conditions, reaching SR values above 91% (Table 2). In the simulated intestinal phase environment, most cultures decreased their populations, reaching values between 46 and 102%. However, all of the tested LAB cultures were able to survive the simulated upper GIT stages.

**Table 2:** Survival of selected 15 lactic acid bacteria (LAB) strains to in vitro gastrointestinal conditions (gastric and intestinal phases)

LAB	Identification	Population (log CFU/mL)*			Survival rate** (SR%)	
		Control (Initial)	Gastric phase	Intestinal phase	Gastric phase	Intestinal phase
*L. casei*	MSI1	6.54 ± 0.00	6.79 ± 0.01	5.76 ± 1.40	103.8	88.1
	MSI5	8.74 ± 0.00	8.79 ± 0.00	6.76 ± 2.13	100.6	77.4
	MRUV1	9.15 ± 0.00^a^	8.80 ± 0.00^a^	7.30 ± 0.40^b^	96.3	79.8
	MRUV6	8.29 ± 0.00^c^	8.79 ± 0.01^a^	8.41 ± 0.02^b^	106.0	101.4
*L. acidophilus*	MVA3	7.71 ± 0.00^c^	7.84 ± 0.00^b^	8.00 ± 0.01^a^	101.7	71.0
*L. nagelli*	MSIV4	8.95 ± 0.00^a^	8.71 ± 0.01^b^	7.90 ± 0.03^c^	97.3	88.3
*L. harbinensis*	MSI3	8.87 ± 0.00^a^	8.77 ± 0.01^a^	5.46 ± 2.20^b^	98.9	61.6
	MSIV2	7.98 ± 0.00^a^	7.84 ± 0.00^b^	4.04 ± 0.08^c^	98.2	50.6
*L. fermentum*	SIVGL1	8.53 ± 0.00^a^	7.77 ± 0.01^b^	4.17 ± 0.06^c^	91.1	48.9
*L. plantarum*	MLE5	8.48 ± 0.00^a^	7.77 ± 0.00^ab^	6.00 ± 1.41^b^	91.6	70.8
	MSI2	7.78 ± 0.00^b^	8.78 ± 0.00^a^	3.98 ± 0.09^c^	112.9	51.2
*P. pentosaceus*	MLEV8	8.26 ± 0.00^a^	7.84 ± 0.00^ab^	7.31 ± 0.56^b^	94.9	88.5
*P. acidilactici*	MSI7	9.00 ± 0.00^a^	8.79 ± 0.00^b^	4.20 ± 0.08^c^	97.7	46.7
*W. paramesenteroides*	MRUV3	6.78 ± 0.00^b^	6.79 ± 0.00^b^	7.98 ± 0.03^a^	100.1	117.7
	MSAV5	7.39 ± 0.00	6.79 ± 0.01	5.25 ± 1.52	91.9	70.8

*Average values ± standard deviations, three independent repetitions; values followed by different letters are significantly different by ANOVA and Tukey (*p* < 0.05); **SR(%) = [log CFU N/ log CFU N0] × 100, where N0 and N are the population values before and after the assay, respectively

As already mentioned in this study, in addition to surviving the gastrointestinal host environment, probiotic bacteria must adhere to the GIT, if beneficial properties are related to the colonization of the host by probiotic LAB. The auto-aggregation ability allows bacteria to persist in the intestinal mucosa and, thus, promote their beneficial effects to the host. LAB co-aggregation is also considered a positive attribute, considering these same strains can manifest effects against pathogens. The auto- and co-aggregation results (Table 3) appeared to be strain-specific.

**Table 3:** Autoaggregation of lactic acid bacteria (LAB) and coaggregation rates between LAB and *L. monocytogenes* Scott A, *E. faecalis* ATCC 19443 and *L. sakei* ATCC 15521 (%), cell hydrophobicity, lactose assimilation, presence of genes associated to beneficial properties tested in 15 selected LAB

LAB	Identification	Autoaggregation (%)	Coaggregation rates (%)			Hydrophobicity (%)	Beneficial related genes						
			*L. monocytogenes* Scott A	*E. faecalis* ATCC 19443	*L. sakei* ATCC 15521		*EF1249*	*EF2380*	*EF2662*	*prgB*	*EFTu*	*map*	*mub*
*L. casei*	MSI1	68.9	61.6	53.5	55.9	99.8	–	–	–	–	+	+	–
	MSI5	73.6	71.5	49.1	56.6	99.9	–	–	+	–	+	–	–
	MRUV1	70.0	54.0	52.3	33.7	98.6	–	–	+	–	+	+	–
	MRUV6	68.7	58.9	52.4	56.8	99.1	–	–	+	–	+	–	+
*L. acidophilus*	MVA3	69.4	69.6	55.8	59.9	99.4	–	–	+	–	+	+	–
*L. nagelli*	MSIV4	63.4	67.4	60.6	65.3	99.3	–	–	–	–	+	–	+
*L. harbinensis*	MSI3	78.7	64.0	52.6	65.6	99.6	–	–	–	–	+	–	–
	MSIV2	71.1	64.3	66.4	48.3	98.8	–	–	+	–	+	+	+
*L. fermentum*	SIVGL1	62.9	65.5	49.7	55.8	97.3	–	–	–	–	–	–	–
*L. plantarum*	MLE5	91.7	62.8	57.1	64.1	97.2	–	–	–	–	+	+	–
	MSI2	86.9	57.8	50.9	57.4	99.5	–	–	–	–	+	–	+
*P. pentosaceus*	MLEV8	96.3	72.7	49.7	62.4	97.1	–	–	–	–	–	+	–
*P. acidilactici*	MSI7	78.9	60.4	59.6	63.0	98.4	–	–	+	–	+	+	–
*W. paramesenteroides*	MRUV3	50.0	57.9	46.2	58.0	98.8	–	–	–	–	+	+	–
	MSAV5	67.4	62.1	55.0	63.1	96.2	–	–	–	–	+	+	–

*** Coaggregation test in plates: *Weissella paramesenteroides* with *E. faecalis*: 13 mm of inhibition halo (the only culture)

Cell surface hydrophobicity is the ability of bacteria to present interactions with mucosal cells. Differences in the cell surface hydrophobicity result from the variation in the level of expression of cell surface proteins among strains of a species and are also due to environmental conditions, which affect the expression of surface proteins. All tested strains showed a high hydrophobicity (96–100%, Table 3).

The identified genes linked to beneficial potential in the tested strains are summarized in Table 3. The genes *EF1249, EF2380* and *prgB* were not detected in any of the tested isolates, while *EFTu* was evident in 13 strains, *map* in 9 strains, *EF2662* in 6 strains and *mub* in 4 strains.

All 15 investigated LAB strains recorded a high ability to grow on MRS agar plates containing 0.5% (*w*/*v*) sodium salts of TC, TDC, GC and GDC (data not shown). On ONPG discs, strong β-galactosidase activity was seen for only five LAB strains, including *L. casei* MSI1, *L. casei* MRUV6, *L. plantarum* MLE5, *L. fermentum* SIVGL1 and *L. nagelli* MSIV4 (data not shown). Among them, *L. nagelli* MSIV4 was negative for the β-galactosidase activity in the API ZYM kit (as mentioned above). Nonetheless, 13 strains had strong β-galactosidase activity according to the API ZYM results. The API ZYM kit is more sensitive than other tests and, consequently, more cultures are positive in that assay than other methods that assess β-galactosidase activity.

## Discussion

MRS-V medium has a good potential to be applied in the isolation of LAB with beneficial potential. The presence of vancomycin is important to inhibit several other bacteria in the screening process. M Colombo, AEZ Oliveira, AF Carvalho and LA Nero [6] previously applied this medium to isolate and select LAB from different origins. The intrinsic vancomycin resistance of some species is due to their specific cell wall characteristics. Thus, MRS-V becomes an option to select probiotic cultures that possess this feature [19]. We isolated eight LAB strains from silage (*L. casei* MSI1 and MSI5, *L. nagelli* MSIV4, *L. harbinensis* MSI3 and MSIV2, *L. fermentum* SIVGL1, *L. plantarum* MSI2 and *P. acidilactici* MSI7), three from cow rumen (*L. casei* MRUV1, *L. casei* MRUV6 and *W. paramesenteroides* MRUV3), two from cow milk (*L. plantarum* MLE5 and *P. pentosaceus* MLEV8), one from cow vaginal mucosa (*L. acidophilus* MVA3) and one from cow oral mucosa (*W. paramesenteroides* MSAV5), respectively. Previous studies demonstrated the presence of LAB with probiotic potential in the dairy environment [20, 21]. From our knowledge, the current report is the first to isolate *L. casei* and *W. paramesenteroides* from cow rumen.

Although the in vitro results for resistance to low pH survival, this behavior is strain-specific (Fig. 1). These findings agreed with those of CG Vinderola and JA Reinheimer [22], regarding the greater tolerance of probiotic bacteria to low pH than other LAB. A García-Ruiz, D González de Llano, A Esteban-Fernández, T Requena, B Bartolomé and MV Moreno-Arribas [23] noted that *Lactobacillus* and *Pediococcus* strains were capable of surviving at low pH values. To the best of our knowledge, we are the first to investigate in vitro the pH and bile resistance of *W. paramesenteroides.* Moreover, all 15 tested LAB strains resisted the bile concentrations typically found in the intestine (Fig. 2), corroborating the findings of CG Vinderola and JA Reinheimer [22].

The enzymatic profiles of the *Lactobacillus* strains evaluated were, in general, similar to those reported by other authors [24, 25] The enzymatic activity is important for many functions of the tested cultures. For example, strains with high peptidase but with low proteinase and esterase/lipase activities may be useful in developing body and texture in cheese production and reducing bitterness [25]. β-Galactosidase activity, which is helpful in improving lactose tolerance in the gut, is pivotal for probiotic cultures [26]. Our results demonstrated the production of this enzyme for 13 of the 15 LAB strains. G Arora, BH Lee and M Lamoureux [27] compared the enzymatic profile of 20 *L. casei* strains and indicated the presence of proteinase, peptidase and esterase/lipase activities. The potent peptidase and esterase activities in *Lactobacilli* have been highlighted by their roles in cheese production, like the acceleration of maturation and enzyme modification. Therefore, these results are valuable for both industrial and research purposes. N Tzanetakis and E Litopoulou-Tzanetaki [28] examined *P. pentosaceus* strains by the API ZYM system: leucine and valine aminopeptidase were found in all strains and β-galactosidase, esterase, esterase/lipase and acid phosphatase were detected in most of the strains, as noticed for *P. pentosaceus* MLEV8 (Table 1). However, *N*-acetyl-β-glucosaminidase, β-glucosidase, lipase and cysteine were negative for this strain, unlike the observations of N Tzanetakis and E Litopoulou-Tzanetaki [28]. To the best of our knowledge, we are the first to report the findings of the API ZYM system on *W. paramesenteroides.* Thus, the API ZYM system helped identify and select the 15 LAB strains with beneficial potential.

The results for the tested isolates for the simulated intestinal phase (Table 2) were in agreement with previous studies with LAB. MB Pisano, S Viale, S Conti, ME Fadda, M Deplano, MP Melis, M Deiana and S Cosentino [24] recorded SRs of more than 98% for *Lactobacillus* strains, as observed also by C Caggia, M De Angelis, I Pitino, A Pino and CL Randazzo [29] and KMO Santos, ADS Vieira, FCA Buriti, JCF Nascimento, MES Melo, LM Bruno, MF Borges, CRC Rocha, ACS Lopes and BDGM Franco [12]. V Vidhyasagar and K Jeevaratnam [30] showed that *Pediococcus* strains could survive both the gastric and intestinal phases. We did not find results for *W. paramesenteroides* strains in the literature.

The auto- and co-aggregation results observed for the tested isolates (Table 3), was previously recorded for other strains with beneficial properties [8]. *L. plantarum* MLE5 and *P. pentosaceus* MLEV8 displayed the highest auto-aggregation properties, of 91.7 and 96.3%, respectively. All 15 tested LAB strains demonstrated more than 50% auto-aggregation, and correspondingly, 14 had more than 60%. Fifteen strains showed 50% co-aggregation with *L. monocytogenes* Scott A. Additionally, 11 strains exhibited more than 50% co-aggregation with *E. faecalis* ATCC 19443, while 4 strains presented between 40 and 50%. Co-aggregation with *L. sakei* ATCC 15521*,* which is non-pathogenic, may play a key role in facilitating the presence of this species in the human GIT. *L. casei* MRUV1 did not show good co-aggregation results (33.7%). The other 13 strains showed between 48 and 63%. Thus, all 15 LAB strains showed co-aggregation abilities with the pathogens tested but the degree of co-aggregation varied, depending on the specific strain. Prior literature studies also established that *Lactobacillus* presented a wide range of auto-aggregation of 5–68% [29] and 28.8–87.7% [12], and up to 60% co-aggregation with *L. monocytogenes* [12].

SD Todorov, DN Furtado, SMI Saad, E Tome and BDGM Franco [8] documented that *Lactobacillus* presented low levels of co-aggregation with pathogens (*L. monocytogenes* and *E. faecalis*) and high levels with *L. sakei,* respectively. KW Lee, JY Park, HD Sa, JH Jeong, DE Jin, HJ Heo and JH Kim [31] showed that *Pediococcus* strains possessed strong auto-aggregation phenotypes, ranging between 65 and 69%. In the same study, *Pediococcus* had 24–29% co-aggregation, and *Lactobacillus* presented 16–26% co-aggregation with *E. faecalis* ATCC 29212 [31]. V Vidhyasagar and K Jeevaratnam [30] mentioned that a *Pediococcus* strain exhibited a maximum aggregation of 89%, with clumping of the cells and that this strain effectively co-aggregated (81%) with *L. monocytogenes*. M Anandharaj, B Sivasankari, R Santhanakaruppu, M Manimaran, RP Rani and S Sivakumar [32] reported that a *Weissella* strain showed a maximum auto-aggregation of 79% and a co-aggregation of 68% with *Escherichia coli* MTCC 1089.

Cell surface hydrophobicity is also an important beneficial feature presented by the tested strains (Table 3). A good hydrophobicity has already been mentioned for a *Lactobacillus* strain (70%) [29], *Pediococcus* strains (55–79%) [30] and both *Lactobacillus* (43–79%) and *Pediococcus* strains (51.3%) [8]. Conversely, we did not find any relevant studies for *W. paramesenteroides.*

The presence of the main surface proteins genes can be associated with a high adhesion ability, competitive exclusion of pathogens and adhesion-dependent stimulation of the immune system by probiotic LAB strains [17]. The tested isolates presented a variable results pattern for the tested genes (Table 3). *EF2662, map, mub* and *EFTu* play a key role in the mechanistic action of probiotic cultures: *EF2662* is a novel putative binding protein gene, and it is responsible for recognizing adhesive matrix molecules, facilitating adhesion [33]. *map* and *mub* are mucus adhesion genes that allow adhesion to GIT mucosal cells. *EFTu* is an adhesion-like factor gene that also aids in cell adhesion. Finally, *map, mub* and *EFTu* are up-regulated in the presence of mucus, in proportional to increasing mucus concentrations [17].

LAB that present bile deconjugation capacity are desired for use in probiotic products for human consumption because studies show it is associated with the reduction of serum cholesterol by these cultures [34]. The results for bile salts deconjugation of the tested isolates were similar to those obtained in other studies, who also recorded strong deconjugation efficiencies for *Lactobacillus, Pediococcus* and *Weissella* strains [12, 29, 32]*.*

Production of the β-galactosidase enzyme enables the probiotic cultures to assimilate lactose and minimize lactose intolerance. All 15 LAB strains could assimilate lactose. Besides, eight LAB strains, namely, *L. casei* MSI5, *L. casei* MRUV1, *L. casei* MRUV6, *L. acidophilus* MVA3, *L. harbinensis* MSIV2, *P. pentosaceus* MLEV8, *W. paramesenteroides* MRUV3 and *W. paramesenteroides* MSAV5 showed better assimilation of lactose than glucose. V Vidhyasagar and K Jeevaratnam [30] and KW Lee, JY Park, HD Sa, JH Jeong, DE Jin, HJ Heo and JH Kim [31] published similar results. The findings are valuable for the dairy industry because these cultures will be able to grow in a milk-based environment. Also, these bacteria can endow fermented products with sensorial properties.

The tests carried out in the present study are the basis to identify LAB cultures that potentially can be used to create new functional foods. However, additional requisites must be evaluated for application in a commercial formulation, including LAB safety aspects, their application in a product formulation as starter cultures (viability, multiplication/fermentation in the selected food matrix), the conferred sensorial attributes, consumers acceptability, and in vivo tests to detect survival rates in the gastrointestinal tract. All these steps need to be conducted using validated analytical methods, in order to develop a new functional food that can provide beneficial heath effect [35, 36].

## Conclusions

There is no doubt that probiotics play a significant role in nutrition and human medicine. However, specific research, regarding isolation, characterization, safety and application of these microorganisms in food is still necessary, as are accurate studies on their mechanisms of action in promoting the desired benefits. We confirmed the dairy chain as a potential source of beneficial strains, since the obtained isolates possess promising beneficial characteristics. Therefore, further assays are demanded to access their safety aspects, behavior in food matrix, sensorial conferred attributes, and their survival/colonization in the gastrointestinal tract using an in vivo model.

## Additional file

Additional file 1: **Table S1.** PCR primers used for identification, fingerprinting by rep-PCR and detection of beneficial properties related genes in lactic acid bacteria isolates obtained from a dairy production environment in Brazil. (DOCX 16 kb)
