# Supplementary material for: Beneficial properties of lactic acid bacteria naturally present in dairy production
Source: BMC Microbiol. 2018 Dec 19;18:219. doi: 10.1186/s12866-018-1356-8 (PMC6300030; doi:10.1186/s12866-018-1356-8)
Supplement: Supplementary file 1 — Table S1. PCR primers used for identification, fingerprinting by rep-PCR and detection of beneficial properties related genes in lactic acid bacteria isolates obtained from a dairy production environment in Brazil. (DOCX 16 kb) [file 12866_2018_1356_MOESM1_ESM.docx]

Supplementary Table 1. PCR primers used for identification, fingerprinting by rep-PCR and detection of beneficial properties related genes in lactic acid bacteria isolates obtained from a dairy production environment in Brazil.

| Target | Sequence gene | Function | References |
| --- | --- | --- | --- |
| *Rep-PCR* | GTGGTGGTGGTGGTG | Differentiation | [Dal Bello *et al.* (2010)](#_ENREF_9) |
| *RAPD PCR* | OPL-01: GGCATGACCT  OPL-02: TGGGCGTCAA  OPL-04: GACTGCACAC  OPL-05: ACGCAGGCAC  OPL-14: GTGACAGGCT  OPL-20: TGGTGGACCA | Differentiation | [Todorov *et al.* (2010)](#_ENREF_30) |
| *16S rRNA* | 8F: CACGGATCCAGACTTTGATYMTGGCTCAG  1512R: GTGAAGCTTACGGYTAGCTTGTTACGACTT | Sequencing | [Felske *et al.* (1997)](#_ENREF_13) |
| *EF1249* | F: GCGGTCGACAAACGAGGGATTTATTATG  R: CTGGCGGCCGCGTTTAATACAATTAGGAAGCAGA | Fibrinogen binding protein | [Fortina *et al.* (2008a)](#_ENREF_14) |
| *EF2380* | F: GCGGTCGACGACATCTATGAAAACAAT  R: TCCGCGCCGCCTTAAACTTTCTCCTT | Membrane-associated zinc metalloprotease | [Fortina *et al.* (2008a)](#_ENREF_14) |
| *EF2662* | F: GGCGTCGACCACTTAAACTGATAGAGAGGAAT  R: CGCGCCGCAATTAATTATTAACTAGTTTCC | Choline binding protein | [Fortina *et al.* (2008a)](#_ENREF_14) |
| *prgB* | F: GCCGTCGACTCGAGGAGAATGATACATGAAT  R: CCTGCGGCCGCGTCCTTCTTTTCGTCTTCAA | Surface protein | [Fortina *et al.* (2008a)](#_ENREF_14) |
| *EFTu* | F: TTCTGGTCGTATCGATCGTG  R: CCACGTAATAACGCACCAAC | Adhesion-like factor | [Ramiah *et al.* (2007b)](#_ENREF_26) |
| *map* | F: TGGATTCTGCTTGAGGTAAG  R: GACTAGTAATAACGCGACCG | Mucus adhesion genes | [Ramiah *et al.* (2007b)](#_ENREF_26) |
| *mub* | F: GTAGTTACTCAGTGACGATCAATG  R: TAATTGTAAAGGTATAATCGGAGG | Mucus adhesion genes | [Ramiah *et al.* (2007b)](#_ENREF_26) |
